# Seeing the forest or the tree depends on personality: Evidence from process communication model during global/local visual search task

**DOI:** 10.1371/journal.pone.0284596

**Published:** 2023-04-21

**Authors:** Sixtine Lefebvre, Virginie Beaucousin

**Affiliations:** 1 Psychologist, PCM Trainer, PCM R&D Projects, Croisy-sur-Eure, France; 2 Université de Rouen Normandie, UR7475, CRFDP, Rouen, France; Georgetown University Medical Center, UNITED STATES

## Abstract

In everyday life, we are continuously confronted with multiple levels of visual information processes (e.g., global information, the forest, and local information, the tree) and we must select information that has to be processed. In the present study, we investigated the relation between personality and the ability to process global and local visual information. Global precedence phenomenon was assessed by a standard global/local visual search task used in many visuo-spatial precedent studies, and the 77 participants were also presented with the standard Process Communication Model (PCM) questionnaire. Results suggest that the ability to process global and local properties of visual stimuli varied according to the Base type of participants. Even if four among six Base types (Thinker, Persister, Harmonizer and Promoter) presented a classical global visual precedence, the two other Base types (Rebel and Imaginer) presented only an effect of distractors and an effect of global advantage, respectively. Taken together, these results evidenced that each human being does not equally perceive the “forest” (global information) and the “tree” (local information). Even if objectively presented with similar visual stimuli, individual responses differ according to the Base, an inter-individual variability that could be taken into account during daily life situations.

## Introduction

Seeing the forest or seeing the tree? In everyday life, we are continuously confronted with multiple levels of visual information processes (e.g., global information, the forest, and local information, the trees) and we must select information that has to be processed. Over the last century, psychologists understood how attentional resources are distributed during visual scene perception [1]. To study global and local processes, Navon used hierarchical stimuli, consisting of large forms (the global level) composed of a suitable arrangement of small elements [1–3]. These stimuli are particularly clever to study global/local processes, because this experimental material included a global level that could be apprehended independently of the local level, and vice-versa. During paradigms that used hierarchical stimuli, two very reproductible effects have been found: a global advantage, characterized by faster global processing than local processing, and a global interference, characterized by the influence of global information during local processing [1, 4]. These effects, defined as the global precedence phenomenon, appear to impact the neural processing very fast, as suggested by the fact that the human brain is sensitive as soon as 150 ms to “the forest before the tree” impression [5]. It has also been showed that global precedence phenomenon is not a predefined way of processing: it evolves during developmental period in children, with an evolution of visual perception mode characterized by a change from a preference for local visual information to an adult-like preference for global information, with a transition in visual preference occurring around 6 years of age [6–8], suggesting that these processes are sensible to both brain maturation and environment influences [6, 9]. A large majority of published studies have focused on experimental stimuli and task variations that could affect the global precedence phenomenon [10–13]. For instance, factors such as the sparsity between local elements [14], the saliency of the global form [15], the exposure duration [16] or the visual angle of presentation [17] modulate the global precedence phenomenon. Some studies have also investigated how interindividual characteristics could affect global and local processes. Indeed, as a matter of fact, even if global and local information present in the visual environment are identical for all of us, the way in which participants process a visual scene varied according to culture [18], gender and age [19], verbal/visual styles [20], field-dependency characteristics [21], and handedness [22]. For instance, Nisbett and Masuda [18] evidenced that when people from different cultures were asked to described a visual scene, Western culture participants focused in a stronger way on local objects in comparison to Asian culture participants, who focused more on the global context of the scene. Similarly, it has been suggested by Poirel and collaborators [21] that individual sensitivity toward global information is related to his/her degree of field dependency [23], defined as the sensitivity to the Gestalt laws of perceptual organization in natural grouping elements (e.g., proximity, similarity, good form and simplicity, that represents the tendency to group together things that are close in space, that are similar, to organize things as simple as possible, respectively [24, 25]). It suggests that even if the global precedence effect seems to be a standard way of processing, in agreement with biological models of visual perception [26, 27], inter-individual variabilities such as social and environment factors affect the way different people perceive their visual world. Even if it has been shown that personal characteristics correlated with neurophysiological processes during mental tasks such as reasoning [28], to our knowledge the relationship between the global precedence phenomenon and interindividual personality structure has never been investigated. Here, we used Process Communication Model® (PCM) questionnaire, elaborated by Kahler, who defined six Personality Types, each being present in everyone [29], with a predominant Personality Type called “Base”. Originated for NASA astronaut selection and training in 1978 (see e.g. [30]), PCM allows defining each person into 6 different Personality Types: Thinker, Persister, Harmonizer, Rebel, Imaginer, Promoter. Each type has its character strengths. The character strengths that belong to: the Thinker type are “Responsible, Logical and Organized”, the Persister type are “Dedicated, Observant and Conscientious”, the Harmonizer type are “Compassionate, Sensitive and Warm”, the Rebel type are “Spontaneous, Creative and Playful”, the Imaginer type are “Reflective, Calm and Imaginative”, the Promoter type are “Adaptable, Charming and Persuasive” [29]. Every person has the six Personality Types, each type being represented at different strengths. The Personality Structure is represented by a Condominium, in which a person’s Base Type is the ground level, the most developed one, the easiest and the most accessible and with the strongest Character Strengths. Every Type is linked to a dominant Perception. There are six Perceptions by which we experience, interpret, and respond to our environment, the one the most developed is the one of our Base. Harmonizer Base perceives the word throughout Emotions. Thinker Base perceives the word throughout Thoughts. Rebel Base perceives the word throughout Reactions. Persister Base perceives the word throughout Opinions. Imaginer Base perceives the word throughout Inaction (Reflexions). Promoter Base perceives the word throughout Actions.

In the present study, we investigated the relation between the Base type and the ability to process global and local visual information. Global precedence phenomenon was assessed by a standard global/local visual search task used in many visuo-spatial precedent studies [31–34], and each participant was also presented with the standard Process Communication Model (PCM) questionnaire. We hypothesized that because of dominant Perception preferences (see above, and see [35]), participants, according to their Base Type, should be differently sensible to global precedence phenomenon during visuo-attentional task. For instance, Rebel Base (who show Spontaneous, Creative and Playful Character Strengths) and Persister Base (who show Observant, Conscientious and Dedicated Character Strengths) should be more sensible to interference effects during visuo-spatial processing than Imaginer Base (who show Calm, Reflective and Imaginative Character Strengths). Thus, the present work will allow to uncover how the global precedence phenomenon is modulated by Base type during visuo-spatial task that is essential in everyday life situations (e.g., [36]).

## Methods

### Participants

A total of 77 healthy volunteer participants (42 women, mean age = 40.75 years ± 8.39 years, Table 1) participated in the experiment. An a priori power analysis using G*Power 3.1 [37] was conducted with a mixed 6x3x4 design with one between-subject factor of group (Thinker Base, Persister Base, Harmonizer Base, Rebel Base, Imaginer Base, Promoter Base) and two within-subject factors (level of target occurrence: local, intermediate, global; number of distractors: 0, 1, 3, 5) indicated that a sample size of 30 participants (5 per group) would be sufficient to detect a medium effect size (f = .25) with a power of .80 and an alpha of .05. All of the participants have normal or corrected-to-normal vision. No participants reported neurological or neuropsychiatric disorders or the use of psychoactive medications. All participants provided written informed consent in accordance with the Declaration of Helsinki (BMJ 1991; 302:1194). The whole procedure was approved by the local ethics committee (CCE n°2022-09-A 2022/10/20).

**Table 1:** Sociodemographic and cognitive characteristics of the participants.

Base	Women/Men	Age	Onward digit span	Backward digit span	Raven matrix	Stroop
Rebel	8/7	37±7	6.4±1.3	5.1±1.4	20.6±2.7	154±129
Thinker	6/10	45±7	6.8±1.5	5.3±1.6	21.6±2.6	183±131
Harmonizer	16/5	40±7	6.7±1.5	5.2±1.4	20.0±2.7	140±115
Imaginer	2/6	45±8	6.6±1.1	5.9±1.2	22.6±1.8	123±71
Persister	4/3	42±11	6.9±1.2	6.0±1.4	22.5±3.6	71±104
Promoter	7/3	38±9	6.1±1.3	5.4±1.8	20.2±3.6	129±85

This Table 1 provides for each type of base (Rebel, Thinker, Harmonizer, Imaginer, Persister, Promoter) the number of women and men, the mean age with its standard deviation (SD), the mean score and the SD for the digit span (WAIS-III), the mean score and the SD for the backward digit span (WAIS-III), the mean score and the SD for the Raven progressive matrix test, and the mean interference score and the SD for the Victoria Stroop task.

### Experimental procedure

**Fig 1:**
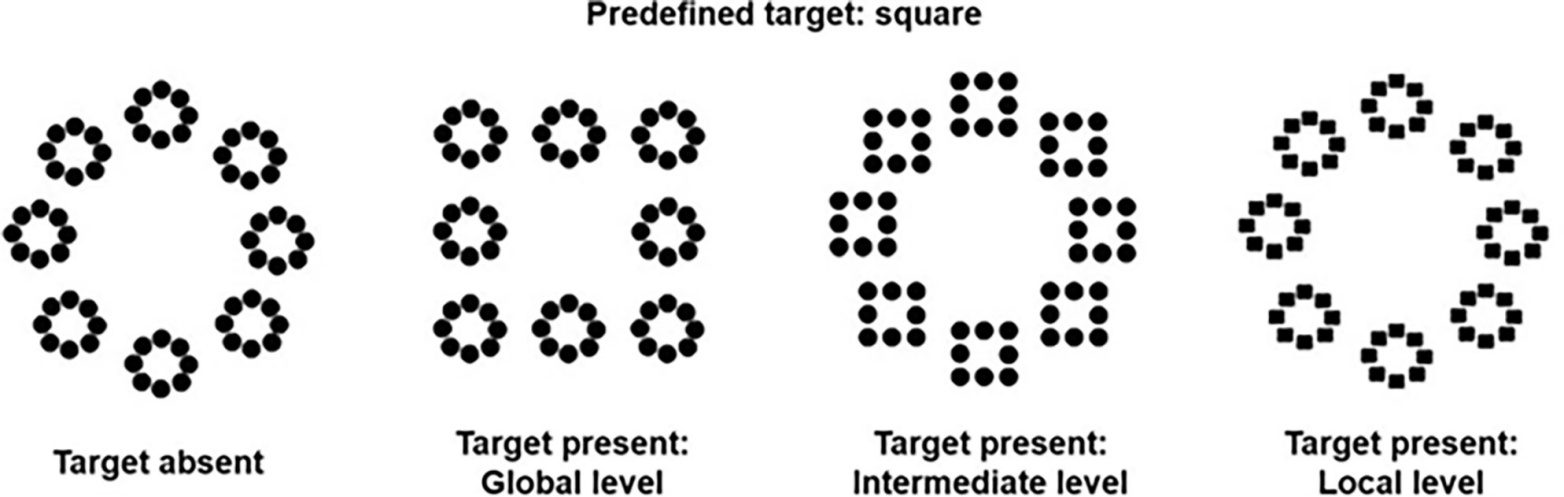
Hierarchical stimuli used in the experiment.

**Fig 2:**
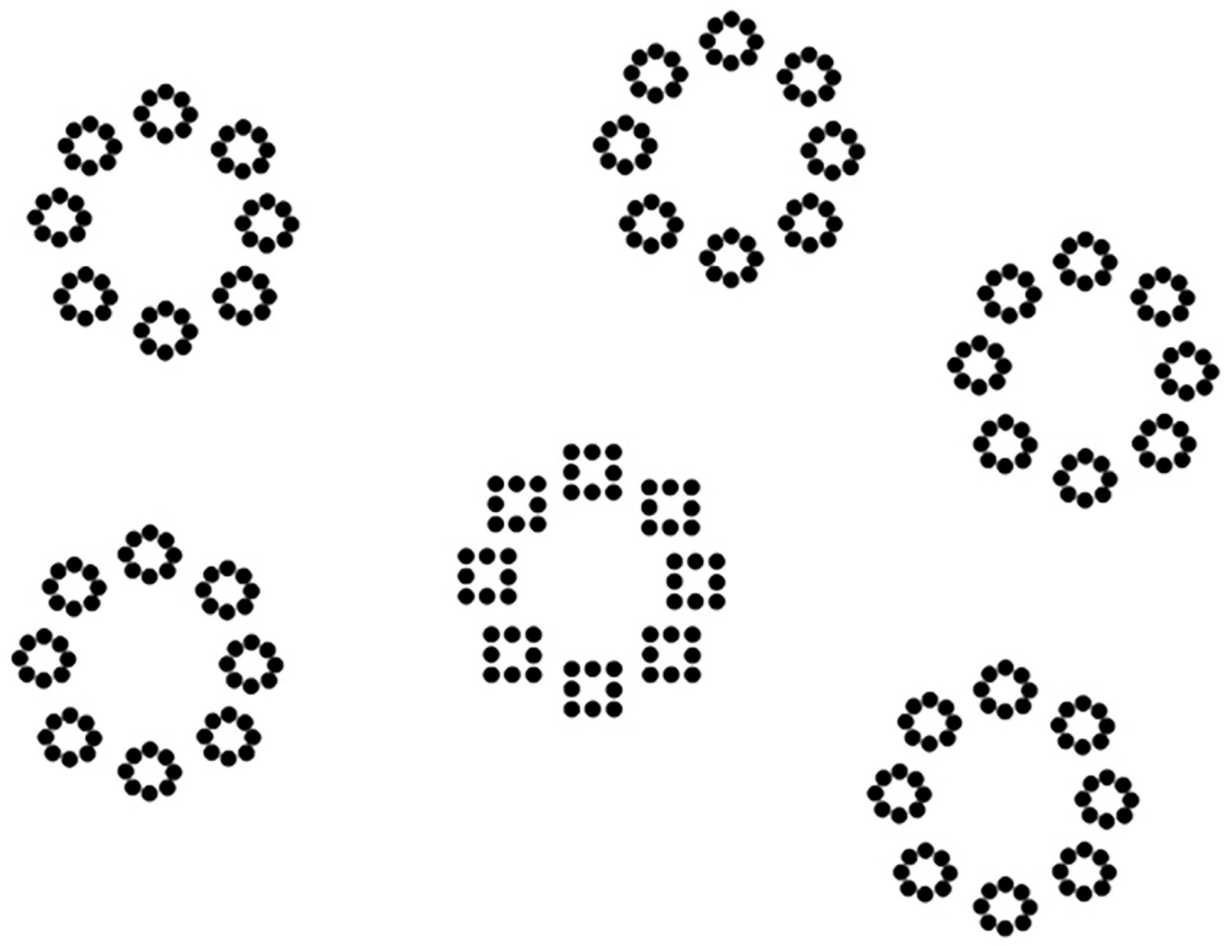
Example of present-target trial with a target present at the intermediate level with five distractors. Note that targets could appear equally often at the global level, the intermediate level or the local level, and there could be zero, one, three or five distractors in the display.

The standard Process Communication Model (PCM) questionnaire was filled by each participant at home in order to have all the time required to complete it. Then the participants were individually presented with a global/local visual task, a working memory digit span task (WAIS-III), a Victoria Stroop task and Raven progressive matrix test in an unique session (Table 1). The global/local task consisted in the presentation of three-level hierarchical stimuli composed of geometrical forms at each level (i.e., global, intermediate, and local, see Fig 1, [9]). Participants had to decide as fast as possible whether a square was present at any level of the hierarchical figure and responded by pressing the left button of the mouse to respond “square present” and the right button to respond “square absent”. The target was actually present in half of the trials. One, two, four or six three-level hierarchical stimuli were presented at the same time on the screen. In the present-target trials, only one hierarchical stimulus contained the target, which appeared at only one level (global, intermediate or local; Fig 2). Thus, in present-target trials, there could be zero, one, three or five distractors. Note that the ratio between the number of targets and the number of distractors was kept constant regardless of the level at which the target was presented. In the absent-target trials, there was no square target: circles were presented at all three levels.

The global/local visual search task was presented using a laptop computer with a 15-inch screen (refresh rate: 60 Hz) running the E-Prime 2 software application (Psychology Software Tools). The participants viewed the stimuli at a distance of approximately 60 cm. Each of the local elements fits within the confines of virtual rectangles of 0.27° in height and 0.20° in width. Intermediate geometric figures were 1.08° in height and 0.81° in width, and global figures were 4.84° in height and 3.62° in width. Present-target items and absent-target items appeared equally often in each virtual quadrant of the screen.

Each participant started with a training session consisting of 16 trials and were instructed to respond as accurately and as quickly as possible. The participant then performed two blocks of 48 trials with 24 present-target trials (6 trials without any distractors, 6 trials with 1 distractor, 6 trials with 3 distractors and 6 trials with 5 distractors; see Fig 2) and 24 absent-target trials in each block (6 trials per number of hierarchical figures appearing on the screen: 1, 2, 4 or 6 hierarchical figures). The trials were randomized within blocks. In the present-target trials, the target appeared equally often at the global, intermediate and local levels. Each trial started with the presentation of a blank screen (500 ± 250 ms), and then a stimulus was displayed. The stimulus remained on the screen until the participant provided an answer. Response times (RTs) were recorded from the onset of the stimulus to the button-press.

Participants ability to stock and manipulate information in short term and in working memory was assessed with onward and backward digit span test. In this task, the participants listened to series of discrete digits and were asked to recall the series of digits in the first part of the task in the same order of presentation, and in the second part of the task in the reverse order of presentation. The participants first performed two series of two digits. The series of digits were incrementally increased by one digit every two trials. The task was stopped when the participants failed to recall two trials with the same number of digits. The short-term memory score was defined as the number of series correctly recalled during the onward part of the task and the working memory score was defined as the number of series correctly recalled during the backward part of the task. The inhibitory control ability was assessed with a Stroop task, in which participants were required to indicate the color of the ink with which the word is written, not to read the written word, as soon as the words appear on the screen. Twenty-four words were presented with congruent information (i.e., ink color congruent with the written word, e.g. BLUE written with a blue ink) and 24 words were presented with incongruent information (i.e., ink color was incongruent with the written word, e.g. the word RED written with a blue ink). Individual interference scores were computed, by subtracting the congruent RTs from the incongruent RTs for each participant. Finally, the Raven progressive matrix test was presented individually to each participant. In each of the 26 trials, they had to identify the missing element that completes a pattern. The score was calculated as the number of trials correctly completed.

## Results

Among all participants, PCM questionnaire indicated that 16 participants were identified having a Thinker Base, 7 had a Persister Base, 21 had a Harmonizer Base, 15 had a Rebel Base, 8 had an Imaginer Base and 10 had a Promoter Base (Table 1). Regarding the global/local task, the present-target trials and the absent target trials, as well as accuracy rates and RTs, were analyzed separately (see the supplementary information in the S1 Table). Because participants were highly accurate and presented a ceiling effect during global/local visual task (mean accuracy ± standard error: 95.8 ± 0.9, 98.9 ± 0.9 and 95.3 ± 0.9 for global, intermediate and local present-target trials, respectively; 99.5 ± 0.4, 98.7 ± 0.4 and 98.7 ± 0.4 for global, intermediate and local absent-target trials, respectively), only RTs were analyzed using Jamovi software application (cc 4.0). Post hoc comparisons were performed using paired *t* tests with Holm-Bonferroni correction.

For the present-target trials, RTs for correct responses were included in a three-factor repeated-measures analysis of variance (ANOVA) with a between-subject factor of group (Base: Thinker, Persister, Harmonizer, Rebel, Imaginer, Promoter) and 2 within-subject factors (the level of target occurrence (global, intermediate or local) and the number of distractors (0, 1, 3 or 5, see the supplementary information in the S1 Table)). For the absent-target trials, RTs for correct responses were included in a similar analysis of variance as those for present-target trials, providing that stimuli were presented at the same location during present- and absent-target trials, but with no target presented during the target-absent trials. Finally, all ANOVAS included onward and backward working memory digit span scores, Stroop task score and Raven progressive matrix score as covariates.

**Fig 3:**
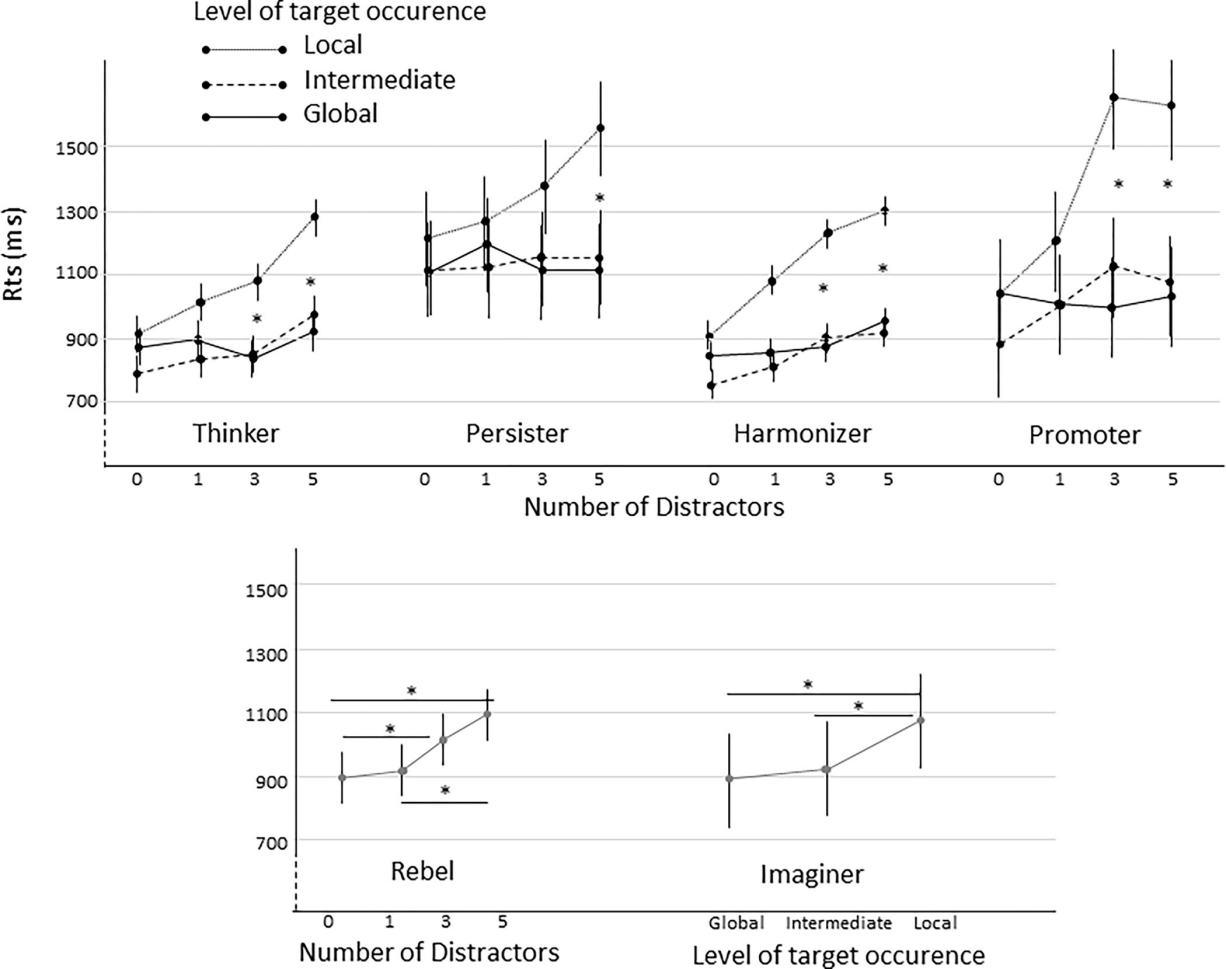
Interaction between the group of participants (Thinker Base, Persister Base, Harmonizer Base, Promoter Base), the level of target occurrence and the number of distractors (top) and effect of the number of distractors and of the level of target occurrence for the Rebel Base and Imaginer Base, respectively (bottom). *p < .05, error bars indicate standard error of the mean.

For present-target trials, the repeated-measures ANOVA revealed main effects of level of target occurrence, *F* (2,134) = 4.51, *p* = 0.013, η_p_^2^ = .06, and number of distractors, *F* (3,201) = 2.93, *p* = 0.035, η_p_^2^ = .04. Regarding level of target occurrence, global and intermediate levels did not differ (*p* = 0.59) and were processed faster than local level (*p’s* < 0.001). The main effect of number of distractors revealed a general RTs increasing with number of distractors present on the screen (all *p’*s > 0.03). Present-target trials RTs were not affected by covariables (onward and backward working memory digit span scores, Stroop task score and Raven progressive matrix score, all *p*s > 0.27 for all main effects and interactions). Finally, this analysis also revealed a significant group x level of target occurrence x number of distractors interaction, *F* (30,402) = 1.60, *p* = 0.026, η_p_^2^ = .11. As shown in Fig 3, group x level of target occurrence x number of distractors interaction revealed that Thinker, Persister, Harmonizer and Promoter Base participants presented a classical global precedence effect, characterized by similar RTs between global and intermediate levels that were not affected by the number of distractors, and slower RTs for local level which were affected by the number of distractors. In agreement with the aforementioned global precedence effect, post hoc analyses revealed level of target occurrence x number of distractors interactions for Thinker Base, *F* (6,90) = 5.25, *p* <0.001, η_p_^2^ = .26, Persister Base, *F* (6,36) = 2.75, *p* = 0.026, η_p_^2^ = .34, Harmonizer Base, *F* (6,120) = 3.72, *p* = 0.002, η_p_^2^ = .16, and Promoter Base, *F* (6,54) = 5.89, *p* <0.001, η_p_^2^ = .40. On the other hand, Imaginer Base and Rebel Base did not present such classical global precedence patterns of responses (*F* (6,42) = 1.30, *p* = 0.277, η_p_^2^ = .16 and *F* (6,84) = 1.82, *p* = 0.104, η_p_^2^ = .12 for Imaginer Base and Rebel Base, respectively). Imaginer Base were only characterized by a global advantage, *F* (2,14) = 7.13, *p* = 0.007, η_p_^2^ = .51, whereas Rebel Base presented only an effect of the number of distractors irrespective of the level, *F* (3,42) = 3.22, *p* = 0.032, η_p_^2^ = .19 (Fig 3).

**Fig 4:**
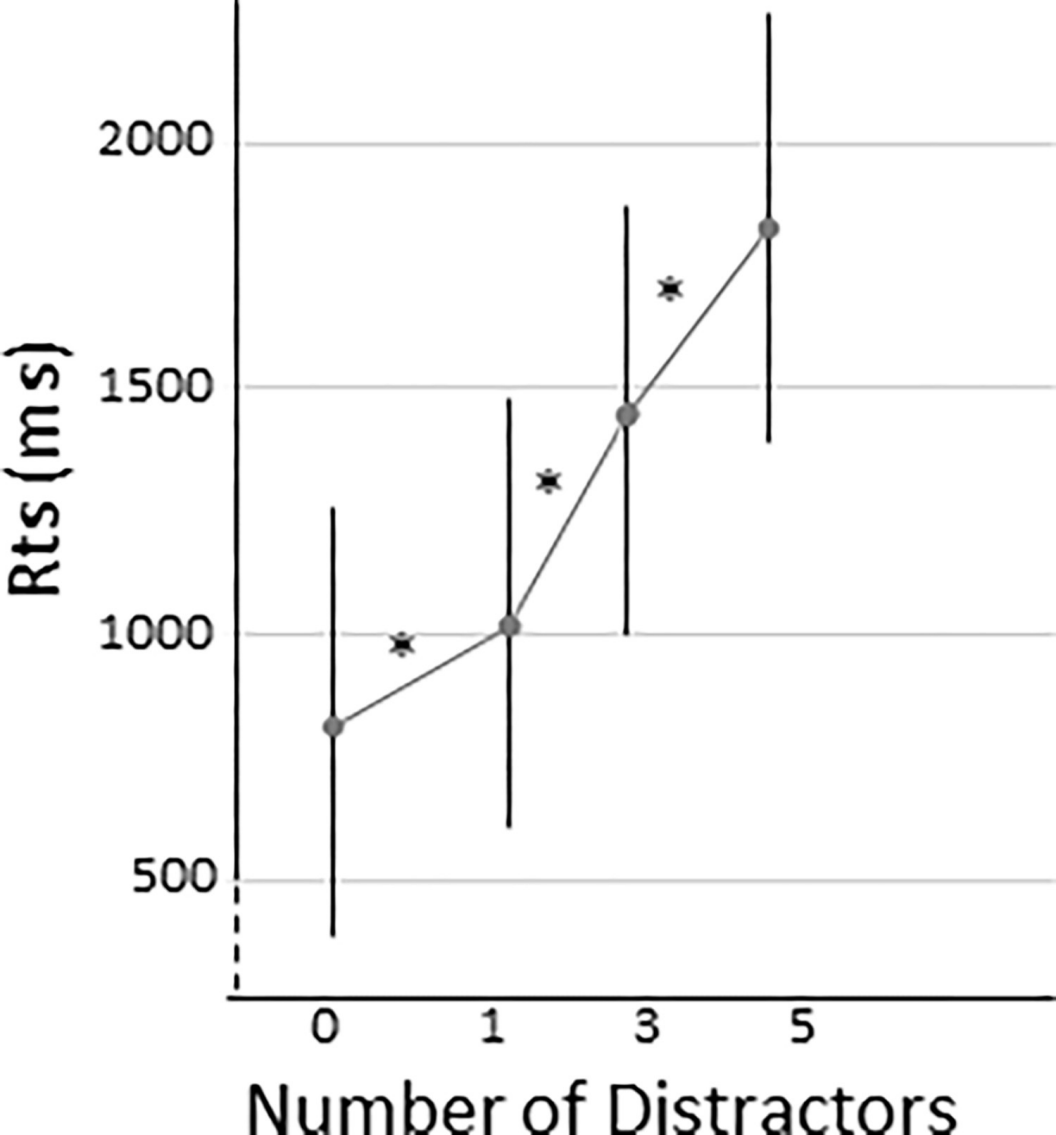
Effect of the number of distractors in absent-target trials. *p < .05, error bars indicate standard error of the mean.

For absent-target trials, the repeated-measures ANOVA revealed only a main effect of number of stimuli present on the display, *F* (3,201) = 6.88, *p* <0.001, η_p_^2^ = .09, and no groups x number of stimuli interaction, *F* (15,201) = 1.62, *p* = 0.07, η_p_^2^ = .11. As shown in Fig 4, RTs increased with the number of stimuli present on the display, whichever group. Finally, absent-target trials RTs were not affected by covariables (onward and backward working memory digit span scores, Stroop task score and Raven progressive matrix score, all *p*s > 0.11 for all main effects and interactions).

## Discussion

Over the last century, psychologists have tried to understand how attentional resources are distributed in a visual display (e.g., [5, 9, 33, 38, 39]). Seminal publication by Navon evidenced the well-known “global precedence phenomenon” [1] characterized by a processing advantage of the global over the local information and an interference from the global information during local processing [1, 4]. The present study investigated how the global precedence phenomenon varied according to the personality structure. For the first time, to our knowledge, experimental results suggest that the ability to process global and local properties of visual stimuli varied according to the Base type of participants. Even if four among six Base Types (Thinker, Persister, Harmonizer and Promoter) present a classical global visual precedence, the two other Base Types (Rebel and Imaginer) presented only an effect of distractors and an effect of global advantage, respectively. This result is coherent with the traditional main result evidenced in the literature: the majority of participants presents a global precedence phenomenon. On the other hand, our findings suggest that a fine-grained analysis revealed that among Bases some participants are either less sensitive to the number of distractors presents on the screen (i.e., Imaginer Base participants) or less sensitive to the level of processing (i.e., Rebel Base participants). This is in line with the view that inter-individual particularities may affect the way we consider visual information [21]. Why do Imaginer Base and Rebel Base participants did not present a traditional global precedence phenomenon compared to other participants? Imaginer Base are described as imaginative, calm and reflective. In agreement with these characteristics, it seems conceivable that Imaginer Base participants consider visual information in a “coarse to fine” way of processing [40], from the global to the local information, with a less competitive way of processing regarding distractors information. In agreement with this hypothesis, Imaginer Base were thus only sensible to the level of processing, with a lower influence of the number of distractors present on the display. On the other hand, Rebel Base participants were only sensible to the number of distractors presented on the display. Rebel Base are described as Playful, Creative and Spontaneous, these particularities may lead to a variation of attentional resources involvement during global and local processes, principally focused on all information presented on the display. Consequently, Rebel Base may be more sensible to the variation of the number of stimuli present on the display. Further investigation will be required to confirm these assumptions, for instance using brain imaging techniques such as functional magnetic resonance imaging or event-related potential methods, in order to investigate the brain network variations subtended the present behavioral results. It seems conceivable that the involvement of both frontal (control processing, see e.g., [41]), associated to parietal and occipital regions (visuo-attentional network, see e.g., [6, 42]) involvement during global and local processes varied according to participants’ Base. Critically, one could argue that because every person has all the six Personality Types, each type being represented at different strengths, the present global/local visual task may evaluate only the participant’ ability to use his/her Thinker level (i.e., Thinker level may represent the logical way of processing in each Personality Structure), rather than a general impact of his/her predominant Base Type. Complementary analyses revealed that neither Thinker level (between 1 and 6) nor percentage of Thinker abilities affected the present results (all ps > .10), ruling out this possibility.

Recent work evidenced that performances on Navon’s hierarchical stimuli relates systematically to the ability to process common real objects [36] and numerous studies argued that global and local processes could be linked to the efficiency of a wide range of abilities such as reading, memory processing, social cognition [43–46] and even decision making and reasoning efficacy [47]. These subsequent cognitive processes were thus proposed to depend on how participant combine local and global information. It seems highly possible that because Base affects the primary consideration of visual information, the following cognitive processes may be modulated by this inter-individual characteristic. The question of potential qualitative and quantitative variations regarding these cognitive abilities should be carefully examined in future studies as well as with more daily life situations.

Similarly, it has been suggested that varying methods approaches during academic learning according to personality aspects of the students might be essential to delivering curricula effectively [48]. The present results go further and reinforce the view that considering the personality information may help understanding how students take into account visual information presented during their schooling. This provides promising clue that will be useful in future studies to optimize learning process.

A limitation must be pointed out here. To the best of our knowledge, this study is the first to link visual process and to personality structure. Even if the sample size of the present study was sufficient to show medium effect size, the present results required to be replicated with a larger sample size. Future studies will also need to explore other cognitive functions in order to better understand the link between cognition and the different personality structures.

In conclusion, the present study evidenced that each human being does not equally perceive the forest (global information) and the tree (local information). Even if objectively presented with similar visual stimuli, individual responses differ according to the Base. Taken together, the current behavioral data suggests that according to the Base, adults differently consider the “forest and the tree”, a variation that has to be taken into account during daily life situations. These findings reinforce the view that personality structure has a strong impact on how we perceive, and probably even think, about the visual world.

## Supporting information

S1 Table Data set. This S1 Table provides for each participant, his/her base (column B: Rebel, Thinker, Harmonizer, Iaginer, Persister, Promoter), his/her age (column C), his/her gender (column D), the score for the onward digit span (WAIS-III, column E), the score for the backward digit span (WAIS-III, column F), the number of the successful Raven progressive matrix test (column G), the interference for the Victoria Stroop task (column BH: the response time (RT) for the incongruent words minus the RT for the congruent words). The rest of the columns (I to BD) provide the accuracy (Acc) and the response time (RT) to the global/local visual search task. (0,1,3,5 correspond to the number of distractors; abs: target absent; global/local/intermediate (interm) correspond to the level where the target is present; pres: target present).
(XLSX)
